# The Occupational Therapy Approach to Addressing Food Insecurity among Older Adults with Chronic Disease

**DOI:** 10.3390/geriatrics4010022

**Published:** 2019-02-15

**Authors:** Lisa A. Juckett, Monica L. Robinson

**Affiliations:** Occupational Therapy Division, 453 West 10th Ave, The Ohio State University, Columbus, OH 43210, USA; monica.robinson@osumc.edu

**Keywords:** food insecurity, occupational therapy, food-related activities, malnutrition

## Abstract

The older adult population is one of the fastest growing age groups in the United States. Various components influence productive aging, and current research has identified nutrition and healthy eating as key factors that impact older adults’ overall health status. While consumption of nutritious meals can help minimize the risk of health decline, the growing rate of food insecurity inhibits older adults’ abilities to access healthy food regularly. Additionally, the high prevalence of chronic disease and disability in older adults can also limit independent participation in food-related activities, such as shopping, self-feeding, and meal preparation. A lack of food access and difficulties engaging in food-related activities place older adults with chronic disease at an increased risk of malnutrition, disability, and losing independence, thereby threatening social participation, healthy aging, and quality of life. Due to their expertise in promoting health and independent living, occupational therapy practitioners may be uniquely positioned to enhance older adults’ healthy eating behaviors through the use of client-centered interventions tailored to food-related activities. This position paper reviews the scope of the occupational therapy profession, the consequences of food insecurity among older adults with chronic conditions, and strategies to enhance food-related activity participation in later life.

## 1. Introduction

The older adult population is one of the fastest growing age groups in the United States [1,2]. Key factors that influence older adults’ health status in later years is nutrition and access to healthy meals [3,4,5]. While older adults’ participation in healthy food-related activities, such as shopping, cooking, and meal preparation, can minimize the risk of disease and disability [6], nearly a quarter of older adults in the United States have experienced marginal, low, and/or very low food security [3,4,7], limiting their ability to secure and consume nutritious meals on a regular basis [8,9] and threatening their quality of life and ability to age successfully [10].

Food insecurity is defined as the insufficient access to the foods needed for people to engage in a healthy and active life [11]. Prior evidence has suggested that food insecurity has been a strong predictor of decreased health in later life and contributes to an increased likelihood of heart disease, cancer, stroke, pulmonary disease, and diabetes [12]. Older adults are already at greater risk for worsening health status than younger age cohorts due to the fact that approximately 65% of older adults are living with two or more chronic conditions [13,14].

In addition to experiencing multiple health conditions [15], food-insecure older adults have also presented with high rates of functional impairments, such as self-care and home management difficulties as well as cognitive deficits [16]. Although federal, state, and local governments have directed funding resources towards public food supports, such as food pantries and the U.S. Supplemental Nutrition Assistance Program (SNAP), there has only been a marginal decline in the level of food insecurity in the United States [11,17]. Furthermore, older adults with functional impairments may need additional assistance beyond financial support, such as transporting, preparing, and cooking food, due to disease-related disabilities or impairments. The profession of occupational therapy specializes in promoting older adults’ independence in these areas and can offer unique insight into the issue of food insecurity among older adults [18]. The aim of this position paper is to describe the distinct role of occupational therapy in addressing food insecurity in the older adult population and to recommend strategies that can assist occupational therapy practitioners in effectively addressing food insecurity and participation in food-related activities with their older adult clients. 

## 2. The Role of Occupational Therapy in Food Insecurity

Occupational therapy practitioners are professionals who assess an individual’s physical, psychological, emotional, cognitive, and/or social conditions and how those conditions may impact his/her ability to engage in occupations. The term “occupation” refers to functional activities such as self-care and home management tasks, work responsibilities, and leisure or social pursuits [19]. The goal of occupational therapy is to maximize individuals’ participation in their day-to-day functioning. Occupational therapy practitioners not only address physical dysfunction, they also consider the psychosocial factors and the individual’s personal interests, behavioral patterns, and functional abilities in the context of their physical, social, and cultural environments. Occupational therapy practitioners can recognize the complexity of food insecurity and address it in a client-centered multifaceted approach. This is supported by Schmelzer and Leto [18] who found that interventions that take into consideration an individual’s personal and environmental contexts can help address the underlying causes of food insecurity, thereby highlighting the distinct value of occupational therapy practitioners. 

When considering the prevalence of chronic disease in older adults, disease-related symptoms and healthcare costs can impact older adults’ functional abilities to shop for food, transport food home, and prepare nutritious meals [20]. The combination of these disease-related consequences can place older adults with chronic conditions at particular risk for food insecurity and subsequent malnutrition, which may further complicate their health statuses. 

Older adults with chronic disease are a vulnerable population who may benefit from occupational therapy interventions to address food insecurity concerns and participation in food-related activities. With regard to the factors that influence food insecurity, occupational therapy practitioners are experts in considering and analyzing the relationship between the person, the environment, and the food-related activities or occupations in which the individual desires to participate [19,21]. When designing occupational therapy interventions, it is considered best practice to turn to theoretical models in order to develop interventions that address key barriers and facilitators to successful participation in occupations. Using the Person–Environment–Occupation (PEO) Model [22], occupational therapy practitioners can identify how food insecurity and health status influence older adults’ independence and engagement in meaningful food-related activities and occupations in later life. 

## 3. PEO Model Application

The distinct value of occupational therapy lies in its comprehensive consideration of factors that influence a person’s level of independent functioning. This notion is exemplified by the PEO model, which recognizes the continuous interactions between the person, their environments, and their occupations and assumes these interactions influence participation in daily activities [22]. *Person* and *environment* factors are all relevant to consider when examining the array of food-related *occupations*. Law and colleagues broadly described occupation in the PEO Model as “activities and tasks done to accomplish a purpose” [22]. Of particular relevance for older adults with chronic disease are food-related activities and occupations such as self-feeding, eating and swallowing, health promotion through nutrition, meal preparation and cleanup, community mobility, finances and financial management, and shopping [19]. The complex relationships between older adults with chronic disease and their environments represent why older adults may be particularly susceptible to food insecurity and have difficulty performing food-related activities and occupations. 

The *person* component of the PEO model is defined as a dynamic being who is constantly interacting with the environment [22]. When assessing the person, occupational therapy practitioners consider a range of skills and characteristics specific to their clients, including but not limited to neuromusculoskeletal functions, gait patterns, psychosocial status, social skills, routines, and roles [19]. All these factors can either support or impede food security and/or engagement in healthy food-related activities. Research has shown impairments in motor, cognitive, and psychosocial function have all been found to limit older adults’ performance in food-related activities, including feeding, dining with others, and preparing meals [10,23,24,25]. 

In addition to appreciating person factors related to food insecurity, occupational therapy practitioners have had a long-standing role in recognizing how the environment can either promote or inhibit participation in occupations, such as meal preparation or shopping [19]. Law and colleagues [22] described the *environment* in the PEO model as physical and social considerations and also recognized the significance of cultural, socio-economic, and institutional aspects of the environment as well. The physical environment, for instance, may refer to an older adult’s home or the neighborhood in which they reside. With regard to the home environment, a recent systematic review suggested home modifications, such as adding task lighting, providing adaptive equipment, and installing assistive technology devices can have a positive impact on kitchen-related food activities, especially when these modifications are implemented by an occupational therapist [26]. Other researchers have suggested occupational therapy interventions can help improve older adults’ independence in daily activities in the home, including in the kitchen space [27]. 

Often, older adults may face built or natural environment barriers (e.g., building accessibility, neighborhood safety, inclement weather, or terrain), which may inhibit their abilities to leave the home. Research has found, for instance, that older adults with greater concerns about their neighborhoods being safe and physically accessible also have a greater incidence of food access challenges [28]. When considering the social aspect of the environment, older adults, particularly those who live alone, who are unable to leave the house, and who lack family support, are at greatest risk for food insecurity and consequential malnutrition [29,30].

The cultural environment should not be overlooked as cultural characteristics can impact older adults’ food security and healthy eating behaviors. Because minority populations are at highest risk for food insecurity, specific cultural considerations should be acknowledged when assessing the eating behaviors of diverse older adults [31]. As one example, although the United States Department of Agriculture (USDA) recommends that daily nutrition intake consist of balanced meals, researchers have gained insight from individuals who are immigrants or refugees and are not as familiar with food choices in their newly-settled country, leading to diets that are not as nutritionally sound [32].

Consistent with the PEO Model, the environment also includes the socio-economic environment and related policies [22]. Story and colleagues [9] suggested that the economic and political environments do not often receive as much attention as the social and physical environments in the literature; however, economic and political contexts play a substantial role in older adults’ food security and healthy eating behaviors. Directly related to the economy, older adults who reside in rural areas, low-income areas, and minority neighborhoods are more likely to lack access to large food stores with healthy food options for purchase [4]. As such, these same low-income, rural, and minority neighborhoods have higher densities of convenience stores, which carry fewer healthy foods and limited quantities of high quality, nutritious produce [33]. Convenience store items are also typically higher in cost than foods found in larger food stores, thereby exacerbating financial strain experienced by older adults who often must budget food expenses due to a limited fixed income [34]. Despite the density of convenience stores in these areas, older adults may not even be able to physically access stores depending on community mobility obstacles and/or perceived safety. 

The Older Americans Act (OAA) Nutrition Programs are positive supports for older adults in both the home and community environments and are designed for older adults who may have challenges participating in food-related activities [16]; congregate and home-delivered nutrition programs have provided meals to older adults over the past five decades; however, these programs have funding restrains that result in reduction of services and long wait lists, especially for home-delivered meal participants. These programs also fall short in recognizing other issues that lead to food insecurity including older adults’ lack of knowledge and skills regarding healthy nutrition, physical inabilities to prepare and eat food, barriers to community mobility, shopping challenges, and difficulties accessing kitchen spaces at home [17,35]. 

Through the lens of the PEO model, occupational therapy practitioners are well-suited to address food insecurity with their older clients in order to enhance engagement in meaningful food-related activities. We recommend practitioners consider adopting the following strategies in order to mitigate older adults’ risk for food insecurity and worsening health status.

## 4. Strategies for Occupational Therapy Practitioners

According to productive aging initiatives from the American Occupational Therapy Association [21], occupational therapy practitioners play an integral role in advancing and promoting wellness and independence of adults in later life. Evidence has indicated that numerous occupational therapy interventions can enhance the quality of life and functional ability levels of older adults [26,27] and may be modified to target the needs of older adults who experience the threat of hunger. As such, occupational therapy practitioners are uniquely positioned to address older adult food insecurity by formally evaluating the older adult and selecting person-, environment-, and occupation-focused interventions as needed. 

Community-dwelling older adults are at a distinct risk for food insecurity [36] as opposed to those who reside in residential or assisted living facilities. Therefore, occupational therapy practitioners who provide community-based services may be in a prime position to integrate basic food security screening/assessment measures into their encounters with older adult clients in order to identify those at risk for food insecurity and who have difficulties performing food-related activities. As one example of the vulnerable, community-dwelling older adult population, home-delivered meal recipients are a group at high risk for experiencing food insecurity and have historically presented with multiple health conditions and health service needs. As depicted in Table 1, results from the 2016 National Survey of Older Americans Act (OAA) indicated that home-delivered meal recipients not only had difficulty participating in food-related activities but also had substantial self-care, home management, and mobility impairments as well [37]. The prevalence of functional impairments and food-related activity difficulties among the home-delivered meal population is one example of why the food-insecure older adult population requires urgent attention from occupational therapy practitioners who provide services in community based settings [2].

## 5. Assessment

According to the USDA, “food insecurity is a complex, multidimensional phenomenon which varies through a continuum of successive stages as the condition becomes more severe” [38]. The use of assessment tools can help identify an individual’s extent of food insecurity, which can be particularly useful during intervention planning. As occupational therapy practitioners recognize the potential for food insecurities within the older adult population, it is well within their current domain to evaluate the impact of food insecurity using generic measures such as the Canadian Occupational Performance Measure (COPM) [39]. The COPM is a client-centered, interview-based assessment that identifies and prioritizes areas of decreased functional performance in daily activities. The occupational therapy practitioner can guide the conversation to also to include food-related occupations in order to establish areas of importance with older clients who may be at risk of food insecurity and potential malnutrition. Additionally, specialized assessment tools can also enhance the evaluation process. For instance, the U.S. Household Food Security Survey Module (HFSSM): Six-Item Short Form [40] may be utilized with older clients who possess food insecurity risk factors. The abbreviated six-item HFSSM serves as a fairly reliable substitution for the full, 18-item HFSSM and was designed to decrease burden on the individuals administering and completing the survey. Data gathered from the HFSSM can be used to assess the status of an older adult’s food security, which ranges from high to very low food security. Because we acknowledge that the lack of familiarity and time restraints may deter occupational therapy practitioners from completing the HFSSM, we recommend that occupational therapy practitioners collaborate and communicate with social workers and/or case managers who may regularly screen for food access concerns within the older adult’s household.

Groups of older adults who experience the greatest risk for food insecurity include those who: are in low income groups, are Black and Hispanic, are divorced or separated, rent property, live alone, live in the South, are under age 75, are unemployed, have less education, and have disabilities [4]. Although these groups possess the greatest risk for food insecurity, screening for food insecurity across all groups can be a valuable action step towards identifying older adults in need of food security and food-related activity interventions. Using the HFSSM is well within the scope of the occupational therapy profession and will help identify food insecure older adults more easily than other screens more typically used in occupational therapy practice.

One recent advancement in occupational therapy measurement has included the development of the Occupational Performance Measure of Food Activities (OPMF) [41]. The OPMF is a 15-item scale which was designed to gather information about clients’ perceived importance, performance level, and satisfaction related to five different food-related activities: shopping, cooking, eating, dining out, and eating healthily. Compared to the HFSSM, the OPMF can provide practitioners with a more client-centered assessment of older clients’ food-related activity performance and may be used in conjunction with the HFSSM to obtain robust detail on the factors that influence food security and food-related activity participation. 

In a participatory action research study on maximizing food resources for those living in poverty, Schmelzer and Leto [17] designed a novel food-related instrument: The Individual Food Resource Profile (IFRP). The IFRP has specific assessment components an occupational therapy practitioner may use to identify issues and develop intervention plans on topics such as current or past use of community food resources, food habits and routines, skill level for managing food resources, dietary preferences and restrictions, and the availability of common kitchen items. 

## 6. Interventions—Occupation

Food-related activities and occupations relevant to older adults include meal preparation/clean up, shopping, self-feeding, eating and swallowing, community mobility, and financial management for food purchases. Community-dwelling older adults have been found to have challenges performing food-related activities and occupations independently, requiring assistance from others in areas such as self-feeding, meal preparation, shopping, and transportation to food stores [23,25,42]. Additionally, older adults have demonstrated poor adherence to food safety practices, as indicated by a lack of refrigerated food storage knowledge, prevalence of expired or rotten food, and difficulties reading expiration labels [43,44]. Occupational therapy practitioners can teach strategies and establish reminders to label food and exercise safe, proper storage practices. Self-feeding may be resolved educating on the use of adaptive equipment such as scoop bowl/plate, built up utensil handles, or a rocker knife [45]; and fall risk interventions may be tailored to address fall prevention in the kitchen and during food-related activities [46,47]. Occupational therapy practitioners can assess and treat eating and swallowing difficulties by addressing oral–motor skills, bolus management, or dysphagia [48]. Teaching financial management and budgeting strategies can also support older adults in their abilities to afford nutritious meals on a fixed income [49]. Lastly, community mobility is multifactorial issue that includes home accessibility, neighbor safety, driving, and alternative transportation. Occupational therapy practitioners can address mobility concerns by installing a ramp or railings on the exterior of the home, identifying age-friendly routes for accessing food stores, teaching an older adult how to use various alternative transportation options, and/or referring an individual for an older driver assessment [50,51].

## 7. Interventions—Person

Research supports that physical, cognitive, and mental health issues all can have a negative impact on food security and food-related activities [4,23]. Occupational therapy practitioners can determine how a physical limitation can impact food-related activities, habits, and routines and develop interventions to increase functional independence. For instance, interventions can be structured to address meal preparation for clients who are wheelchair users, food preparation with one-handed techniques for stroke survivors, and the use of raised color dots on appliances for a person with low vision. For those who have cognitive and mental health impairments, interventions may include providing simple written instructions during meal preparation, sequencing steps when storing food items, reviewing stress and anxiety management strategies when in the community, or educating on the use of basic kitchen technology (e.g., reminders or timers). These interventions may be meaningful for older adults who reside alone as well as for those who have valued roles related to securing and preparing food for others (e.g., spouse, neighbors). 

## 8. Interventions—Environment

Fried and colleagues [52] suggested there is an increased burden both physically and financially on families and the healthcare system when there are inadequate environmental supports for those living in the community who have functional limitations. Home assessments and modifications can help address these concerns and are intended to increase safety, accessibility, and independence for older adults in their homes. Home modifications, for instance, can be implemented in the kitchen to decrease fall risk, improve overall safety with food-related activities, and maximize older adults’ ability remain in their own homes—where the majority of older adults prefer to reside [26]. Specific options for interventions can include adapting the kitchen space to allow for cookware to remain in reach at the shoulder or waist level, installing task lighting to reduce the risk of injury when chopping food or handling hot items, modifying how a cooking task is completed by applying energy conservation techniques, training older adults on the use of adaptive equipment and devices, or adding railings to enter and exit a home after shopping [26]. 

The community-based occupational therapy practitioner is a professional who can also identify social and cultural issues that may impact food security and nutrition. The practitioner may be the first person to identify the need for home-delivered meal services. Occupational therapy practitioners working with community-dwelling older adults may also be able to connect clients to community food resources, such as federally-funded nutrition services supplemented by the OAA. Under the OAA’s nutrition programs, older adults aged 60 and over may be eligible to receive either home-delivered meals or to participate in congregate dining services. Home-delivered meals, often referred to as “Meals-on-Wheels,” were established with the goal of providing nutritional support to older adults in greatest social and economic need [53]. Relatedly, congregate dining services are offered at public community sites, such as senior centers, churches, and recreation facilities, and were designed to foster social interaction among older adults while also providing participants with nutritious meals [16]. By sharing knowledge about these two nutrition programs for older adults, occupational therapy practitioners may be able to serve as a valuable link between older adults and community-based food resources. 

Practitioners are also encouraged to increase their cultural awareness of food preferences of other cultures and educate certain subgroups on healthy alternatives to preferred foods while still respecting cultural backgrounds of older adults who may perceive less nutritious foods to be an integral part of their cultural identity [54]. In particular, practitioners are recommended to assist diverse older clients in identifying healthy meals that are culturally valued, budgeting for food expenses, developing healthy shopping lists, and preparing nutritious meals that are appealing but still align with cultural norms and beliefs [32].

## 9. Conclusions

Novel approaches are needed to address the prevalence of food insecurity that is plaguing the older adult community. Because of their expertise in recognizing the intersections of the person, the environment, and meaningful occupations, occupational therapy practitioners are well-suited to address food insecurity and food-related activity impairments among older adults with chronic disease The strategies provided in this paper align with the tenets of the PEO Model—a frequently applied model in the occupational therapy profession—and can be applied to enhance the health and well-being of the food insecure older adult population. Knowledge of food-related interventions and community-based food resources/services are examples of how occupational therapy practitioners can best meet the needs of older adults with food access and healthy eating concerns. Engagement in food-related activities and occupations has been highly valued for many older individuals, impacting healthy aging and quality of life in later years, and requires urgent attention from occupational therapy practitioners due to the growing population of older adults with chronic disease who experience food insecurity [4,55]. 

## Figures and Tables

**Table 1:** Rates of self-care and home management impairments among home-delivered meal recipients ^a^.

Type of Impairment	(%)
Heavy cleaning (e.g., scrubbing floors, washing windows)	81.1
Walking	65.8
Going outside the home	53.6
Meal preparation	46.8
Light cleaning (e.g., dishes, sweeping)	46.5
Driving	43.4
Bathing	36.8
Moving around inside the home	34.7
Getting in/out of a bed or chair	33.3
Dressing	25.5
Money management	23.5
Medication management	18
Toileting	15.8
Telephone use	9.9
Eating	9.3

^a^*n* = 868. Data drawn from the 2016 National Survey of Older Americans Act Participants: Home-Delivered Meals [37].
